# A New Method for Functional Evaluation of Motor Commands in Patients with Cerebellar Ataxia

**DOI:** 10.1371/journal.pone.0132983

**Published:** 2015-07-17

**Authors:** Jongho Lee, Yasuhiro Kagamihara, Shinji Kakei

**Affiliations:** 1 Movement Disorders Project, Tokyo Metropolitan Institute of Medical Science, Tokyo 156–8506, Japan; 2 Department of Neurology, Tokyo Metropolitan Neurological Hospital, Fuchu, Tokyo 183–0042, Japan; The University of Western Ontario, CANADA

## Abstract

Quantitative evaluation of motor functions of patients with cerebellar ataxia is vital for evidence-based treatments and has been a focus in previous investigations of movement kinematics. Due to redundancy of the musculoskeletal system, muscle activities contain more information than the movement kinematics. Therefore, it is preferable to analyze causal anomalies of muscle activities to evaluate motor functions in patients. Here we propose a new method to evaluate the motor functions at the level of muscle activities and movement kinematics. Nineteen patients and 10 control subjects performed two movement tasks of the wrist joint, a step-tracking task and a pursuit task, with a manipulandum. The movements of the wrist joint and activities of the four wrist prime movers were recorded. We developed a linear model for the wrist joint to approximate the causal relationship between muscle activities and movement kinematics in terms of the wrist joint torque. We used a canonical correlation analysis to verify the causality between the muscle activities and the movement kinematics in the model. We found that the activities of the four muscles were related almost entirely to the position and velocity, with negligible correlation with the acceleration of the wrist joint. Moreover, the ratio of the weights for position- and velocity-related torque components characterized the contents of the muscle activities in terms of the movement kinematics. Next, we compared the ratios for the two movement tasks between the controls and patients. In control subjects, the ratios indicated clear task-specific changes that conformed to the functional requirements of the tasks. In contrast, in patients, the task-specific changes diminished highly significantly. The present results indicate that this ability to accommodate motor commands to the task requirements provides a novel quantitative parameter to characterize motor functions in patients with cerebellar ataxia.

## Introduction

The quantitative evaluation of the motor functions of patients with cerebellar ataxia is essential for identifying evidence-based treatments and monitoring the progress of diseases. In order to achieve this goal, some researchers have developed systems to quantitatively evaluate arm movement; these systems can capture some of the characteristic features of the movement kinematics of cerebellar ataxia, such as slow movement, deficiency of smoothness, and inaccuracy of movement [1–7]. The quantitative analysis of movement kinematics (i.e., descriptions of the movement trajectories) is easier to perform and less demanding for patients. However, it does not provide as much information as analysis of muscle activities (i.e., motor commands) because the former has much fewer degrees of freedom than the latter. For instance, it is impossible to evaluate co-contraction by analyzing movement kinematics, whereas it is easy by analyzing muscle activities. Thus, it is appropriate to analyze muscle activities to understand the generation mechanisms of cerebellar ataxia. In line with this idea, some researchers have qualitatively compared the muscle activities and movement kinematics of these patients [1,8–14]. They suggest that the delayed onset of agonists and antagonists and/or imbalances between the activities of the two account for ataxic movements, such as delays in movement initiation, dysmetria, or an inability to coordinate multi-joint movements. However, these comparisons are largely qualitative and cannot explain cerebellar ataxia in terms of motor control.

To evaluate cerebellar ataxia in terms of motor control, it is necessary to make quantitative analyses of the causal relationships between anomalous motor commands and the resulting abnormal movements. Recently, we developed a simple model that establishes a symmetric relationship between movement kinematics (position, velocity, and acceleration) of the wrist joint and the activities of the four wrist prime movers [15,16]. The symmetric relationship provides a ratio of two fitting parameters, viscosity *B* and elasticity *K*. In the model, values “*B”* and “*K”* can be interpreted as the weights of two torque components that are proportional to the velocity and position of the wrist joint, respectively. Therefore, the ratio may reflect contents of motor command, thereby suggesting what is considered for preparation of motor command [15].

In this study, we calculated the ratio of the fitting parameters “*B*” and “*K*” with a canonical correlation analysis (CCA) between the muscle activities and the movement kinematics. We then tested the above-mentioned hypothesis first in control subjects by comparing the ratios for two wrist movement tasks that require different control strategies. We found that the ratios of fitting parameters “*B*” and “*K*” in the control subjects showed clear task-specific changes that conformed to the functional requirements of the tasks. We also determined the ratios for the two tasks in the patients and compared them with those of the control subjects. We found that the task-specific changes diminished highly significantly in the patient group.

## Materials and Methods

### Subjects

Ten healthy control subjects with no history of neurological abnormalities (3 women and 7 men, 46–71 years old, mean = 58.1 years old, all right-handed; see Table 1) and 19 patients with cerebellar ataxia (12 women and 7 men, 29–77 years old, mean = 60.5 years old, all right-handed; see Table 2) participated in the study. All of the subjects were informed of the purpose and procedures of the experiment in advance and provided written informed consents prior to their participation. The protocol was approved by the ethics committees of the Tokyo Metropolitan Institute of Medical Science and the Tokyo Metropolitan Neurological Hospital and was conducted in accordance with the ethical standards of the Declaration of Helsinki.

**Table 1:** Characteristics of the control subjects.

Case	Age	Sex	*B*_*r*_ */K*_*r*_ ratio for step-tracking	*B*_*r*_ */K*_*r*_ ratio for pursuit
C1	46	M	0.03	1.91
C2	48	M	0.13	1.34
C3	63	M	0.11	1.28
C4	51	M	0.15	1.10
C5	54	M	0.14	1.28
C6	52	M	0.24	1.32
C7	61	F	0.28	1.58
C8	67	M	0.20	1.08
C9	68	F	0.17	1.30
C10	71	F	0.20	0.86

**Table 2:** Characteristics of the patients with cerebellar ataxia.

Case	Age	Sex	Diagnosis	Duration of illness (yrs)	ADL (1–3)	*B*_*r*_ */K*_*r*_ ratio for step-tracking	*B*_*r*_ */K*_*r*_ ratio for pursuit
P1	62	F	MSA-C	1	1	0.20	0.75
P2	77	F	SCD	4	1	0.27	1.17
P3	62	M	SCD	9	1	0.20	0.69
P4	72	M	MSA-C	3	1	0.09	0.85
P5	69	F	SCD	5	1	0.22	0.57
P6	65	F	MSA-C	2	1	0.25	0.37
P7	58	F	SCD	7	1	0.14	0.37
P8	34	F	SCD	9	1	0.32	1.11
P9	69	M	SCD	3	1	0.16	0.20
P10	70	M	SCD	10	1	0.34	0.78
P11	39	F	SCD	9	1	0.10	0.23
P12	65	F	MSA-C	2	2	0.27	0.39
P13	29	F	SCD	9	2	0.27	0.50
P14	76	F	SCD	5	2	0.28	0.27
P15	58	F	SCD	24	2	0.16	0.42
P16	57	M	MSA-C	4	2	0.13	0.56
P17	60	F	MSA-C	9	3	0.31	0.35
P18	71	M	MSA-C	4	3	0.53	0.24
P19	56	M	MSA-C	2	3	0.27	0.38

MSA-C = multiple system atrophy (MSA) with cerebellar features, SCD = spinocerebellar degeneration, ADL = activities of daily living. ADL levels are 1: walks without assistance, 2: uses a walker, 3: wheelchair dependent.

The patients’ clinical data are summarized in Table 2. Activities of daily living (ADL) of the patients were rated 1, 2 or 3 [1]. Patients with ADL = 1 (n = 11) had milder cerebellar ataxia and were capable of living independently by themselves. Patients with ADL = 2 or 3 (n = 8) had more severe cerebellar ataxia and experienced difficulty performing ADL by themselves. We included those patients who were capable of understanding instructions about the two movement tasks and of keeping the cursor within the initial target for three seconds by themselves. Otherwise, we excluded the patients from the present study. In addition, they were cooperative during the experiment.

### Experimental setup and movement tasks

The apparatus and experimental setup were the same as those described in our previous study (see [15] in detail). Briefly, the subject sat on a chair approximately 60 cm in front of a computer screen that displayed a cursor and a target, and grasped a Strick-Hoffman type manipulandum ([17], Hoyo Elemec Co., Ltd., Sendai, Japan) with his/her right hand. The forearm was comfortably supported with an armrest and held in a position midway between full pronation and full supination. The cursor was a black point (ϕ = 0.9°) that moved in proportion to the subject's wrist movements. One degree of wrist movement moved the cursor approximately 2.2 mm on the screen. The center position of the manipulandum corresponded to the center of the screen, and the cursor moved left for flexion, right for extension, up for radial deviation, and down for ulnar deviation. The target was displayed as an open circle with an inside diameter of 4.5° of wrist movement.

**Fig 1:**
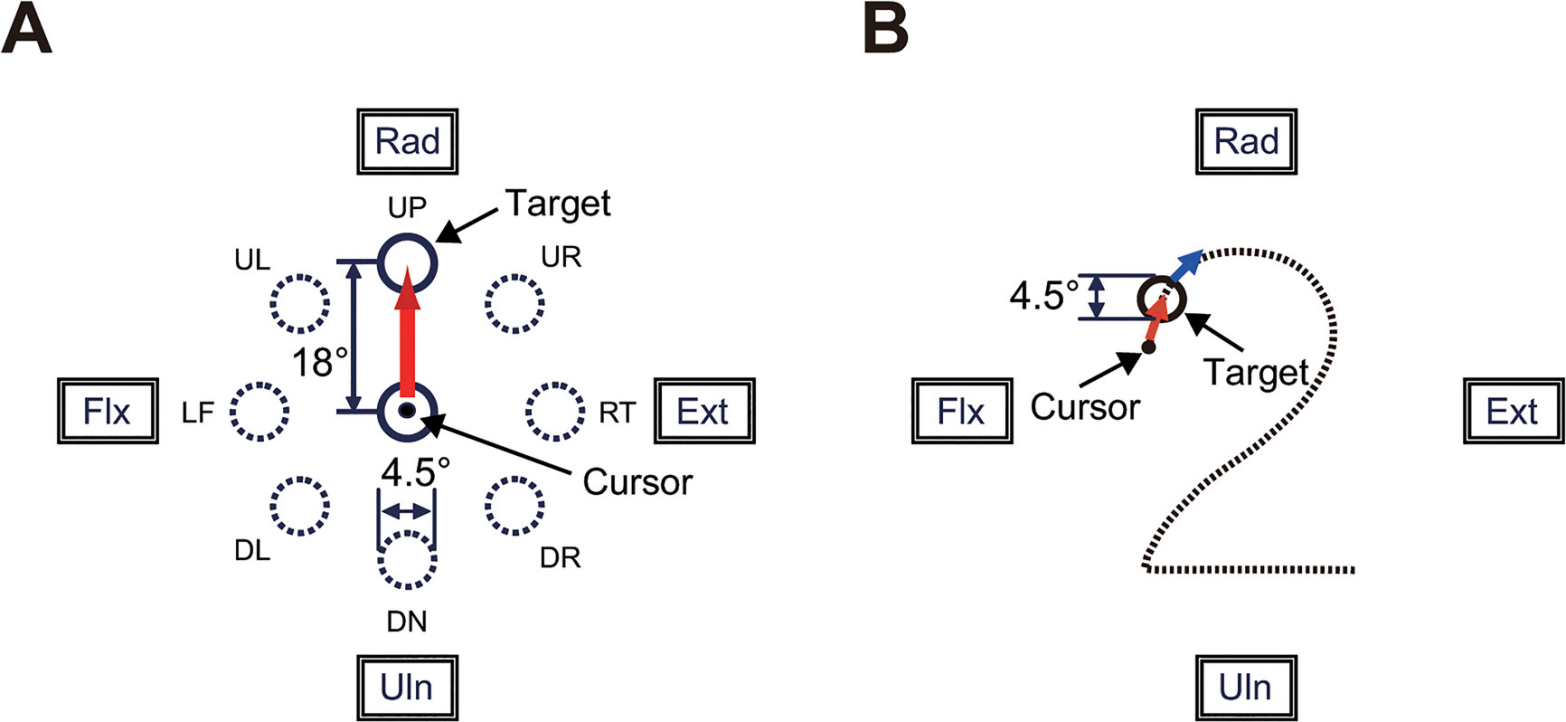
Experimental tasks. (A) Arrangement of targets and required movement of a cursor for the step-tracking task. The target was displayed as an open circle with an internal diameter of 4.5° of wrist movement. The cursor was a black point (ϕ = 0.9°) that moved in proportion to the subject's wrist movements. The position of the cursor in the center target corresponded to the center of the screen, and the cursor moved left for flexion movements, right for extension movements, up for radial deviation, and down for ulnar deviations of the wrist. The subjects were required to move the cursor from the center target to the new target as rapidly and accurately as possible. UP, up; UR, up and right; RT, right; DR, down and right; DN, down; DL, down and left; LF, left; UL, up and left; Rad, radial deviation; Ext, extension; Uln, ulnar deviation; Flx, flexion. (B) The pursuit wrist task. The subject was instructed to maintain the position of the cursor within the target moving along the path of Fig 2 at a constant speed (6.2°/s).

We asked the subjects to perform two movement tasks of the wrist joint: the step-tracking task (Fig 1A) and the pursuit task (Fig 1B).

#### 1) Step-Tracking Task

Each subject was asked to perform a step-tracking wrist task in eight directions (Fig 1A, up [UP], up and right [UR], right [RT], down and right [DR], down [DN], down and left [DL], left [LF], and up and left [UL]). To initiate a trial, the subject placed the cursor inside the target, which was positioned at the center (X = 0°, Y = 0°) of the screen. After a variable hold period (1–2 s), the target moved to one of eight targets that were located at a place equivalent to 18° of the wrist joint from the central target. The subject was required to move the cursor immediately to the new target as rapidly and accurately as possible. After another variable hold period to advance to the next trial, the target was moved back to the central position. The target location changed in a clockwise fashion, starting at the UP position and ending at the UL position. After a cycle of practice trials for eight target locations, each subject performed the task three times for each of the eight locations.

#### 2) Pursuit Task

**Fig 2:**
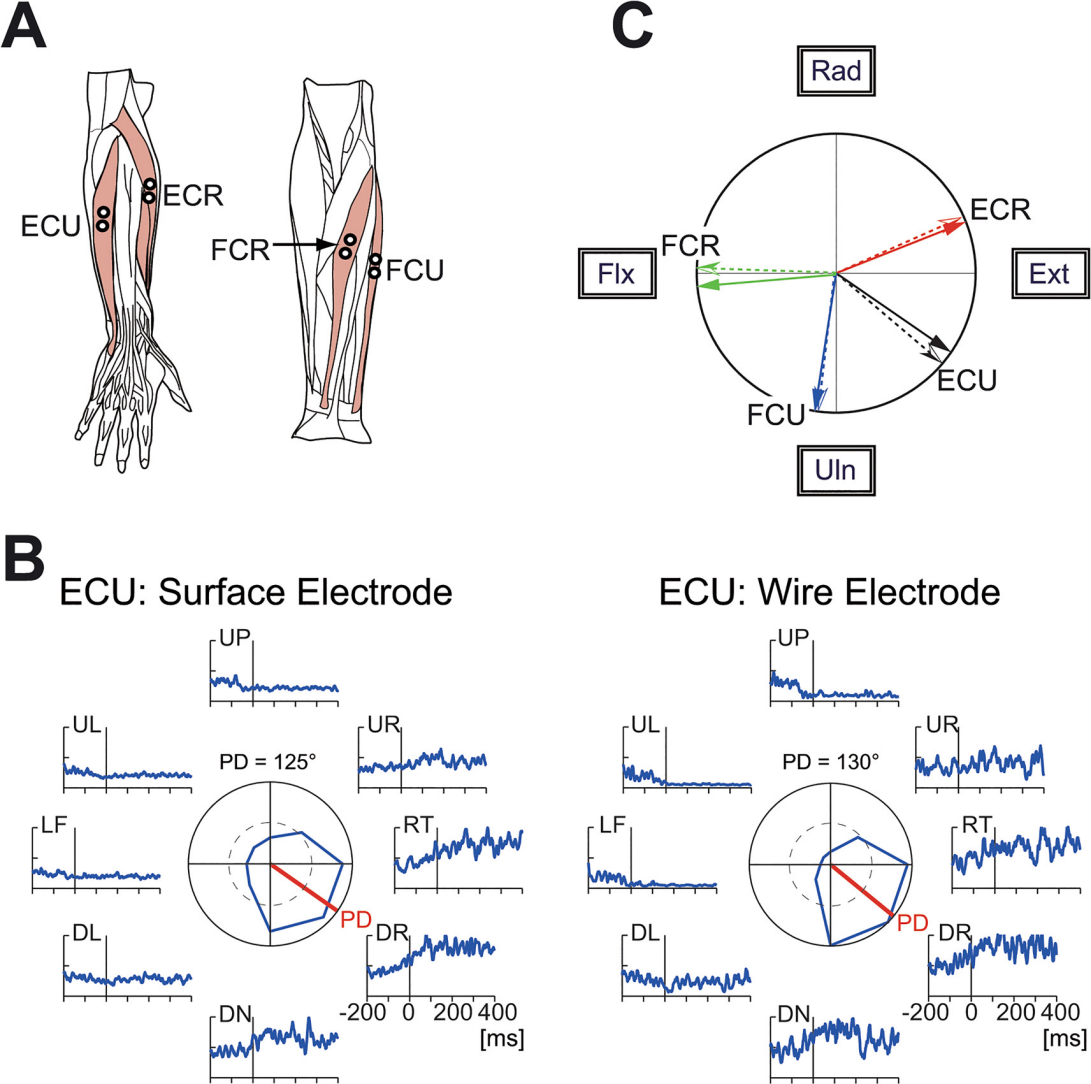
Muscles related to the wrist joint and comparison of surface and wire electromyography (EMG) signals. (A) The four wrist prime movers from which EMG activity was recorded. ECR, *extensor carpi radialis*; ECU, *extensor carpi ulnaris*; FCU, *flexor carpi ulnaris*; FCR, *flexor carpi radialis*. We recorded the activity of the *extensor carpi radialis longus* (ECRL) and *extensor carpi radialis brevis* (ECRB) together as ECR because these two muscles are indistinguishable with surface electrodes. (B) Comparison of the surface and wire EMG activity recorded simultaneously from ECU. The raw EMG signals were filtered (τ = 10 ms) [18], fully rectified, and averaged for 10 trials. The polar plot in the center shows muscle activity within an interval of ±25 ms relative to the movement onset. The red bars in the center indicate the preferred direction, which was calculated with cosine fitting [18]. (C) Correspondence of the preferred direction of EMG activity recorded simultaneously with surface electrodes (solid arrows) and wire electrodes (dotted arrows). ECR surface = 68°, wire = 66°; ECU surface = 125°, wire = 130°; FCU surface = 189°, wire = 187°; FCR surface = 265°, wire = 272°.

Each subject was asked to perform a smooth pursuit wrist movement for a target moving at a constant speed (Fig 1B). To initiate a trial, the subject placed the cursor inside of the target, which was stationary at the upper left (X = -10°, Y = 8°) of the screen. After a hold period of 4 s, the target began moving slowly and smoothly along the path of the Fig 2 at a constant speed (6.2°/s). The subject was required to maintain the position of the cursor within the moving target as much as they could. After three practice trials, each subject performed the task five times. The path is not visible to the subject during the task, while he/she has some knowledge about the motion of the target due to the practice trials.

### Data acquisition

During the task, we recorded the wrist position (X and Y) and electromyography (EMG) signals for four wrist prime movers (*extensor carpi radialis* [ECR], *extensor carpi ulnaris* [ECU], *flexor carpi ulnaris* [FCU] and *flexor carpi radialis* [FCR]). The four muscles were chosen due to the following two reasons. First, they are the wrist prime movers and are essential for wrist movement. Our previous studies showed that only seven forearm muscles were active during step-tracking movements of the wrist joint in monkeys [18,19]. From those seven muscles, we selected the four wrist prime movers by excluding three minor finger muscles. In fact, the combined activity of these four muscles is sufficient to describe movement kinematics at the level of the wrist joint torque in humans [15,16]. Second, it is relatively easy to record their activities with surface electrodes.

The EMG signals were recorded with Ag-AgCl electrode pairs spaced 10 mm apart, amplified (x 100,000) and band-pass filtered (150–30,000 Hz) by an amplifier (AB-611J, Nihon Kohden, Tokyo Japan), and sampled at 2 kHz. The typical locations of the surface electrodes are shown in Fig 2A. The position of each electrode pair was adjusted for each subject to maximize the EMG signals for a specific movement, considering the preferred directions of the four muscles [17]. In addition, the position of each electrode pair was adjusted to maximize the EMG activities for the wrist movements and minimize those of the finger muscles. In order to confirm the effectiveness of the adjustments, we performed a control study with two healthy volunteers (SK and JL). We compared the EMG signals recorded with a surface electrode pair (surface EMG signals; Fig 2B, left) with the signals that were simultaneously recorded with a wire electrode pair inserted directly into the muscle (wire EMG signals; Fig 2B, right). The intramuscular location of the wire electrodes was identified with low-threshold (10–150 μA) evoked twitches for electrical stimulation with the electrodes [17,18]. As shown in Fig 2C, the surface EMG (solid arrow) and wire EMG signals (dotted arrow) were similar for each muscle tested.

The EMG signals were digitally rectified and filtered with a second-order low-pass filter ([20–22], cut-off frequency, 3.0 Hz) to estimate the muscle tensions from the surface EMG signals [20–23]. The tension of each muscle was normalized with a simple normalization technique [23] that sets the amplitude of the muscle tension for 0.78 Nm of isometric wrist joint torque as 1. Finally, we subtracted the normalized muscle tension at the central position from the normalized tension to set the tension of each muscle at the central position to zero. We used the processed muscle tension of each muscle to estimate wrist joint torque.

### Wrist joint model and identification of the relationship between muscle activities and movement kinematics

**Fig 3:**
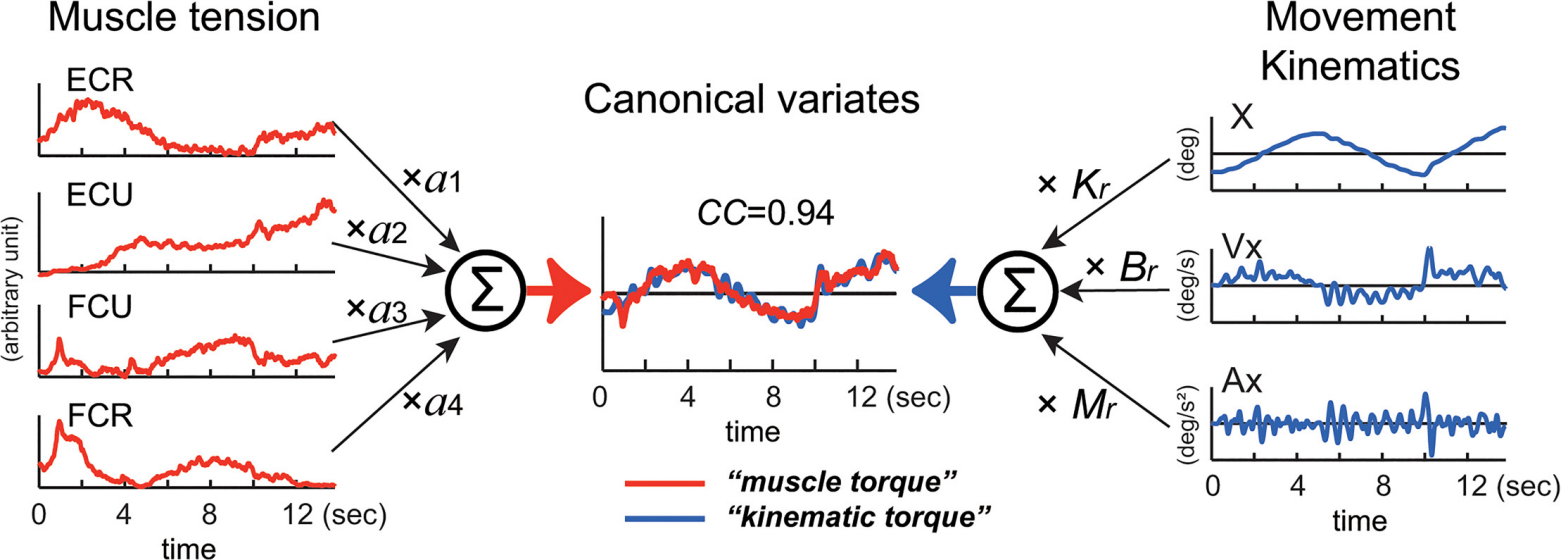
Explanation of the relationship between muscle tension and movement kinematics modeled in Eq (1). The left side represents the middle of Eq (1). EMG activities of the four muscles (ECR, ECU, FCU, FCR) converted into *muscle tension* are linearly summed (∑) after multiplying parameter *a*_*1*_–*a*_*4*_, respectively to obtain *muscle torque* in the center (*red line*) (see Materials and Methods). The right side represents the right side of Eq (1). Acceleration (Ax), velocity (Vx) and position (X) of the wrist joint are summed (∑) after multiplying the inertia parameter (*M*), the viscous coefficient (*B*_*r*_) and the elastic coefficient (*K*_*r*_), respectively to obtain kinematic torque in the center (*blue line*) (see Materials and Methods). We used a canonical correlation analysis (CCA) to obtain values of these parameters. The two canonical variates, *muscle torque* and *kinematic torque*, were calculated by substituting the values for the fitting parameters in Eq (1). Note the high canonical correlation (*CC* = 0.94) between the two canonical variates (in *Estimated torque*). This Fig explains calculation of torque around the x-axis, but the same method applies to calculation of torque around the y-axis.

**Fig 4:**
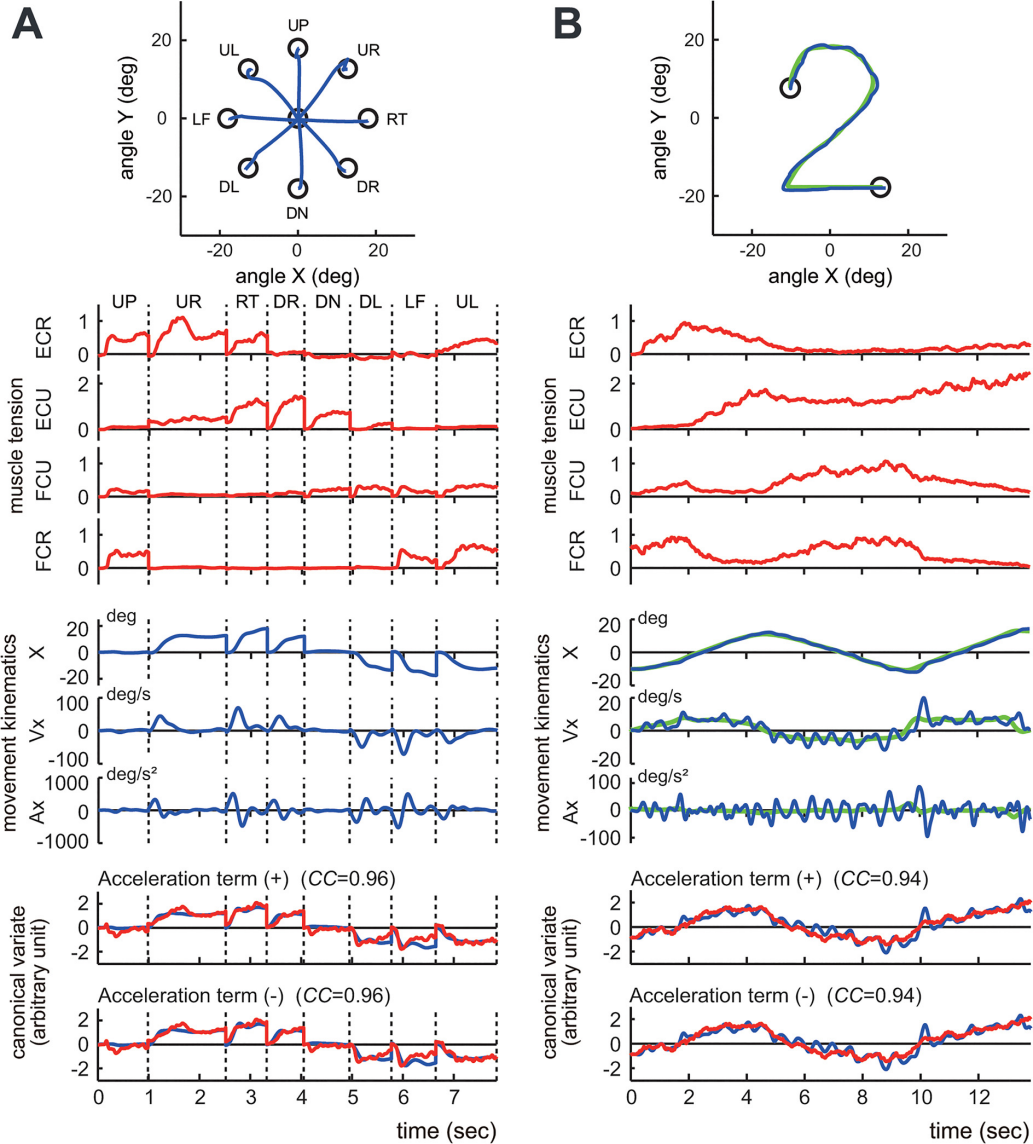
Identification of the relationship between muscle activities and movement kinematics in a control subject. (A) The step-tracking task. (B) The pursuit task. The insets demonstrate the movement trajectories (blue lines) for a single trial. The green line in the right inset indicates the trajectory of the target. The top four traces (*muscle tension*) show the normalized muscle tension of the four wrist prime movers: ECR, ECU, FCU and FCR. The middle three traces (*movement kinematics*) show the horizontal (i.e., x-axis) components of the movement kinematics: angle (X), angular velocity (Vx), and angular acceleration (Ax), respectively. The green lines in (B) indicate the kinematics of the target motion. The bottom panels (*canonical variate*) depict the wrist joint torques estimated from the muscle tension (i.e., *muscle torque*) (red) and the movement kinematics (i.e., *kinematic torque*) (blue). The upper panel (*Acceleration term* (+)) shows the estimated torques that were calculated with Eq (1), which includes the acceleration term. The lower panel (*Acceleration term* (-)) shows the estimated torques calculated with Eq (3), which excludes the acceleration term. The high canonical correlation *CC’s* for the two estimated torques in both (A) and (B) indicate the similarities of the two estimates. Note that the estimated torques without the acceleration term (blue lines in the lower panel) are almost identical to the estimated torques with the acceleration term (blue lines in the upper panel), suggesting a minor contribution of the acceleration term in the identification. The parameters *M*_*r*_, *B*_*r*_, and *K*_*r*_ for the fit in (A) were set as 0.0019, 0.014, and 0.090, respectively, while the same parameters for the pursuit task (B) were set as 0.0019, 0.115, and 0.085, respectively. The damping ratio ζ for the step-tracking task (A) was 0.548, while the ζ for the pursuit task (B) was 4.54.

**Fig 5:**
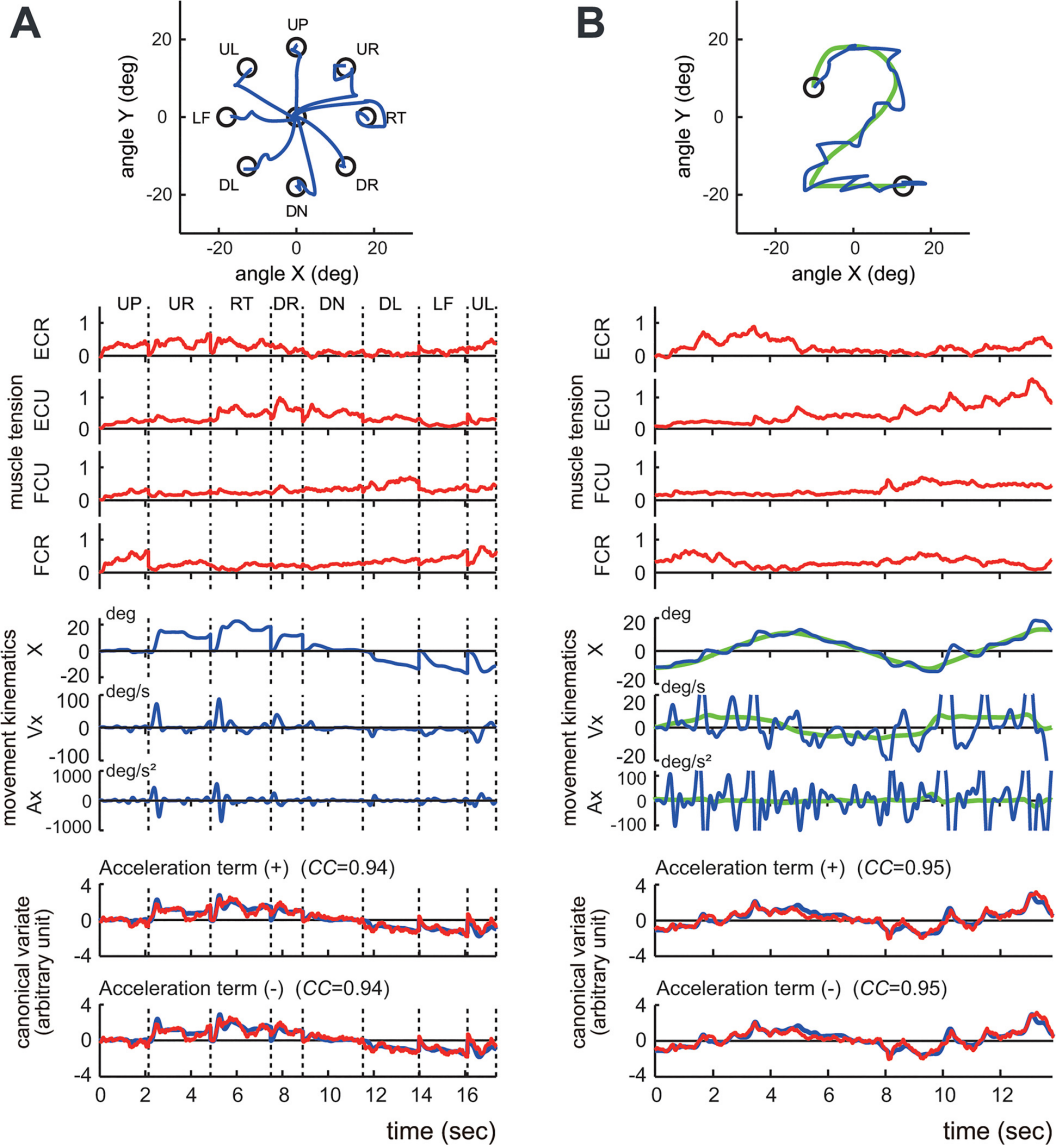
Identification of the relationship between muscle activities and movement kinematics for a patient with cerebellar ataxia. (A) An example of the step-tracking task. (B) An example of the pursuit task. For both (A) and (B), the conventions are the same as in Fig 4. The bottom panels demonstrate the kinematic torques (blue) and the muscle torques (red). The upper panel (*Acceleration term* (+)) shows the estimated torques that were calculated with Eq (1) that includes the acceleration term. The lower panel (*Acceleration term* (-)) shows the estimated torque that was calculated with Eq (2) that excludes the acceleration term. The high *CC’s* for the two estimated torques in both (A) and (B) indicate the similarities of the two estimates in the patient. Note that the estimated torques without the acceleration term (blue lines in the lower panel) are almost identical to the estimated torques with the acceleration term (blue lines in the upper panel), suggesting a minor contribution of the acceleration term in the identification in the patient. The parameters *M*_*r*_, *B*_*r*_, and *K*_*r*_ for the fit in (A) were set as 0.0014, 0.023, and 0.082, respectively, while the same parameters for the fit in (B) were set as 0.0014, 0.030, and 0.114, respectively. The damping ratio ζ for the step-tracking task (A) was 1.054, while the ζ for the pursuit task (B) was 1.198.

We assumed that, if the activity of the four wrist muscles governs the movement kinematics of the wrist joint, it should be possible to reconstruct the wrist joint torque that is calculated from the equation of motion with the activities of the four muscles (Eq (1)).

$$\tau \left(t\right)=\sum_{i=1}^{4}a_{i}T_{i}\left(t\right)=M\ddot{\theta }\left(t\right)+B\dot{\theta }\left(t\right)+K\theta \left(t\right)$$ (1)

where τ(t) denotes the wrist joint torque. *T*_*i*_ represents the tension of each muscle processed as described above (e.g. Figs 3, 4 and 5: muscle tension: ECR, ECU, FCU, and FCR) and *a*_*i*_ denotes the parameters that convert muscle tension into wrist joint torque (see left side of Fig 3). The sign (+ or-) of *a*_*i*_ works as a constraint to limit the pulling direction (i.e., direction of the mechanical action) of each muscle [15,24]. The variables *θ*(*t*), $\dot{\theta }\left(t\right)$, and $\ddot{\theta \left(t\right)}$ represent the angle, angular velocity, and angular acceleration of the wrist joint, respectively. *M*, *B* and *K* are the inertia parameter (kgm^2^), the viscous coefficient (Nms/rad) and the elastic coefficient (Nm/rad).

Eq (1) holds only when there is a high correlation between the wrist joint torque that is estimated with the movement kinematics (*kinematic torque*: right side of Eq (1)) and the wrist joint torque that is estimated with the muscle activities (*muscle torque*: middle of Eq (1)). To identify the relationships between the muscle activities and the movement kinematics for each of the step-tracking and pursuit tasks, we have to find two sets of parameters *a*_*1*_, *a*_*2*_, *a*_*3*_, *a*_*4*_, and *M*, *B*, *K* that optimize the match between the kinematic torque and the muscle torque, for each task. We used CCA [25] for the activities of the four muscles, i.e. (*T*_*1*_(t), *T*_*2*_(t), *T*_*3*_(t), *T*_*4*_(t)), and the movement kinematics, i.e. ($\ddot{\theta }\left(t\right),\dot{\theta }\left(t\right),\theta \left(t\right)$) for each task in each subject with SAS (University Edition, Release: 3.1, SAS Institute Inc. NC, USA). The program yielded two parameter vectors (*a*_*1*_, *a*_*2*_, *a*_*3*_, *a*_*4*_), and (*M*, *B*, *K*) such that the pair of canonical variates (*a*_*1*_, *a*_*2*_, *a*_*3*_, *a*_*4*_)(*T*_*1*_(*t*), *T*_*2*_(*t*), *T*_*3*_(*t*), *T*_*4*_(*t*))^T^ (= $\sum_{i=1}^{4}\;a_{i}\;T_{i}\left(t\right)$) and (*M*, *B*, *K*)($\ddot{\theta }\left(t\right),\dot{\theta }\left(t\right),\theta \left(t\right)$)^T^ (= $M\ddot{\theta }\left(t\right)+B\theta \left(t\right)+K\theta \left(t\right)$) maximize their correlation (i.e. CC) (see Fig 3). In the analysis, we used the “NOINT” option that omits subtraction of means from the data, to keep the muscle activities always positive or zero. As expected, CCs we obtained in the present study were rather high regardless of subjects or movement tasks, as will be described in the Results section. Note that, with CCA, we cannot determine true values of *M*, *B* or *K*, but can obtain their ratios. Therefore, in the following, we use *M*_*r*_, *B*_*r*_, *K*_*r*_ instead of *M*, *B* and *K* to represent that we focus primarily on their ratios. On the other hand, the parameters *M*, *B* and *K* in Eq (1) are assumed to take specific values within physiological ranges. Therefore, we further addressed two issues: a) whether specific values of *B*_*r*_ and *K*_*r*_ provided a better correlation than other pairs of *B*_*r*_ and *K*_*r*_ with the same ratio, and b) how the correlation deteriorated for different ratios of *B*_*r*_ and *K*_*r*_. For each of the step-tracking and pursuit tasks, we optimized the match between the kinematic torque and the muscle torque in the next two steps, using a constrained least mean square method (*fmincon* function of Matlab, Ver. 7.11.0.584(R2010b), Mathworks, MA). The constraint was necessary for the signs of *a*_*i*_ in order to limit pulling directions of the four muscles as described above [15,24].

**Fig 6:**
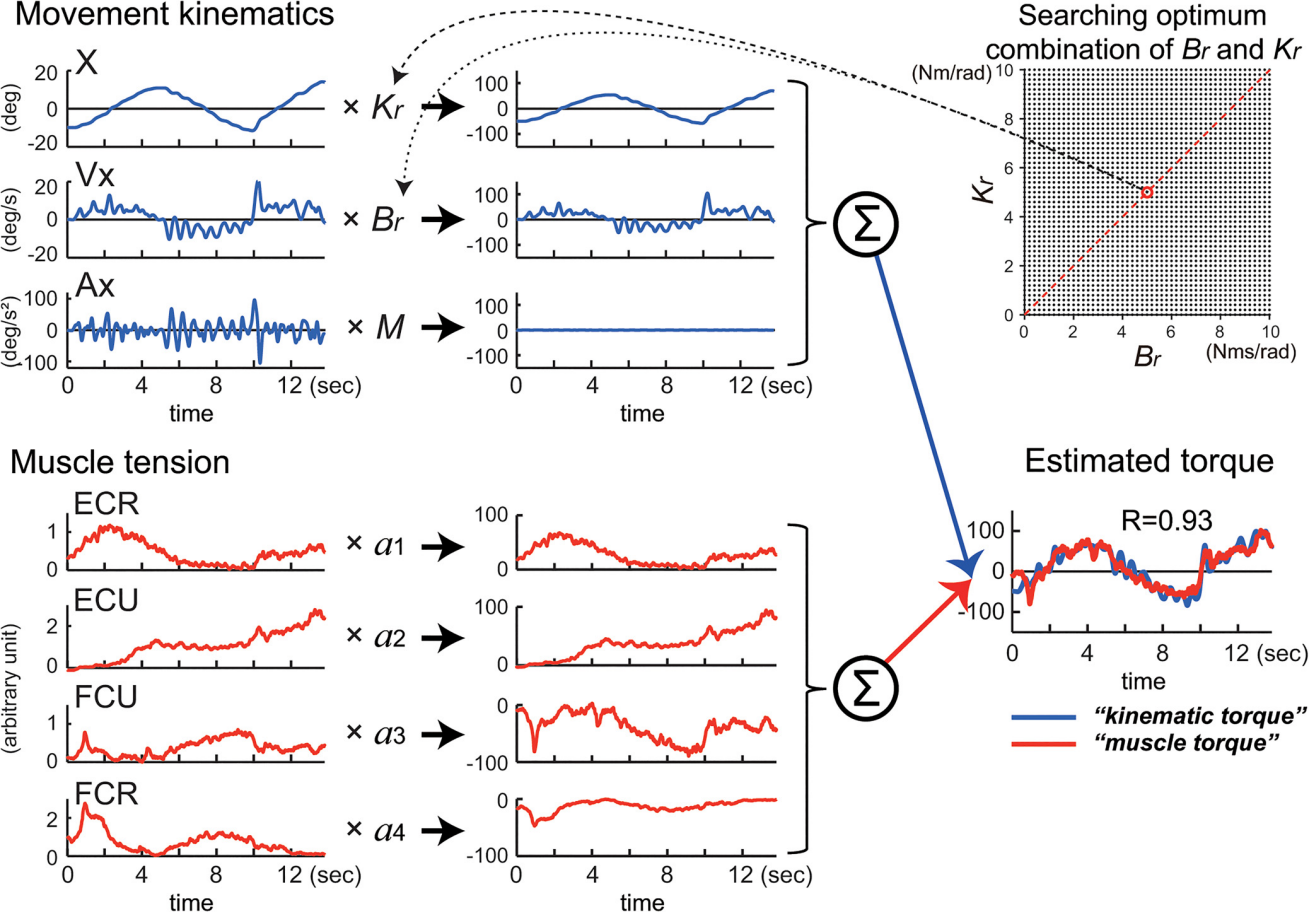
An alternative way to estimate the optimal combination of fitting parameters *B*_*r*_ and *K*_*r*_ for the wrist joint model (1): *searching method*. First, we calculated the inertia parameter *M* for each subject using the Eq (2) and chose a combination of *B*_*r*_ and *K*_*r*_ within the physiological ranges (*upper right inset*) to obtain *kinematic torque* (*lower right*). The average inertia parameter (*M*) for the controls and the patients (n = 29) was 0.0017 ± 0.00036 kgm^2^ (range: 0.0012–0.0023 kgm^2^). Note that contribution of the acceleration term to the *kinematic torque* was almost negligible (see *flat line*). Then we searched for the optimal combination of *a*_*1*_ *-a*_*4*_ that maximized the *R-*value between the *muscle torque* and the *kinematic torque* obtained above (*lower right*). For the regression, we set signs (+ or-) of *a*_*1*_-*a*_*4*_ (*lower left*) in order to limit pulling directions of the four muscles [15,24]. See Fig 7A or 7B for examples of this method. Note the correlation *R* (= 0.93) is almost identical to *CC* (= 0.94) in Fig 3.

*1) Step 1*: For a physiological range of *B*_*r*_ (0 ≤ *B*_*r*_ ≤ 10 Nms/rad, 0.2/step) and *K*_*r*_ (0 ≤ *K*_*r*_ ≤ 10 Nm/rad, 0.2/step) [26–28], we calculated the *kinematic torque* (blue lines of *estimated torque* in Fig 6, *lower right*) using the linear equation of motion, as shown on the right side of the Eq (1) (see Fig 6, *upper left*). Then, as described in the middle of Eq (1), we optimized the match between the kinematics torque and the linear sum of the four muscle tensions (*muscle torque*, red lines of *estimated torque* in Fig 6, *lower right*) by adjusting *a*_*i*_ with the least squares method (see Fig 6, *lower left*).

**Fig 7:**
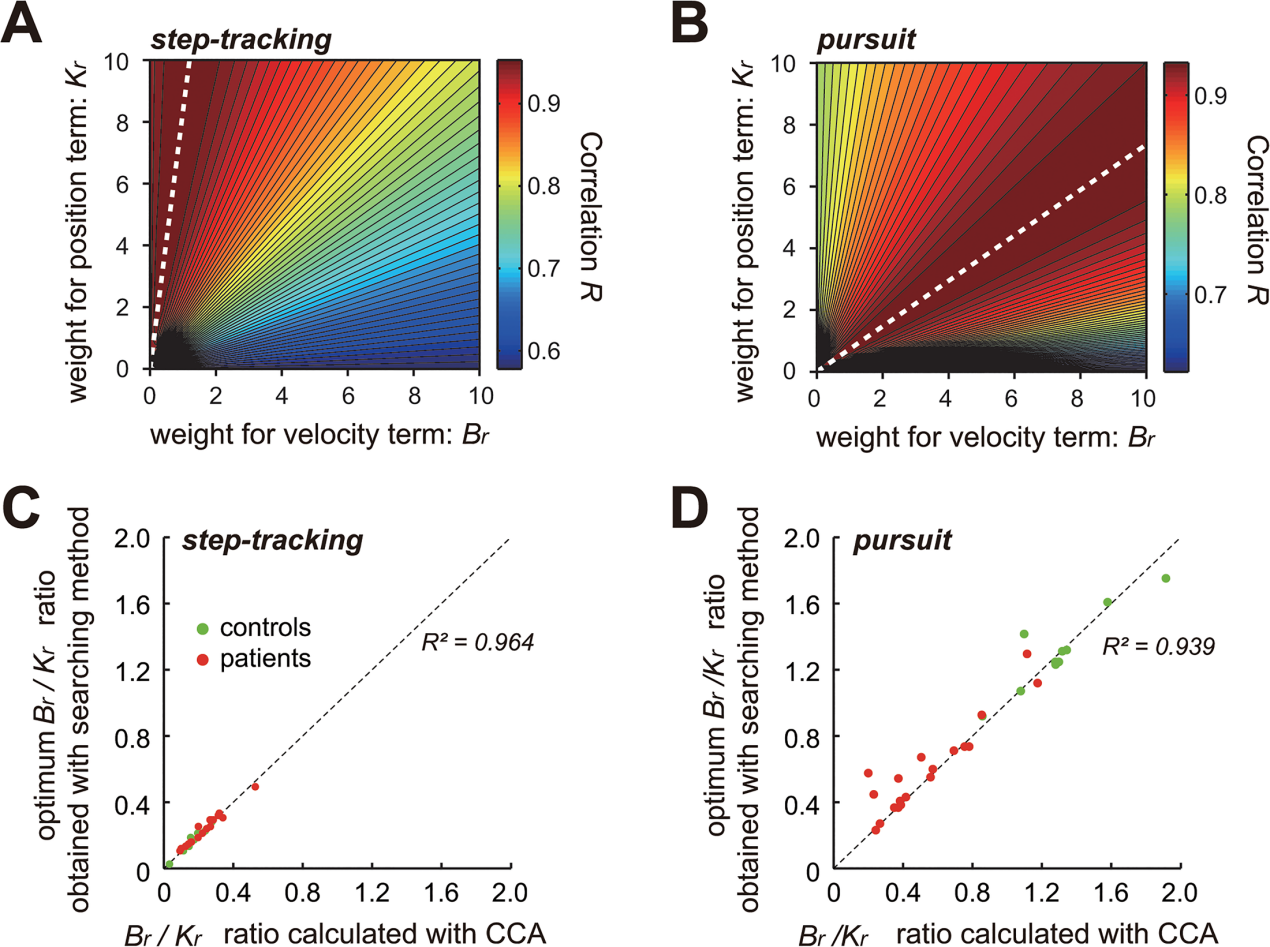
Different *B*_*r*_ */K*_*r*_ ratios for the two movement tasks. Contour plots of the correlation coefficient *R* between the *muscle torque* and *kinematic torque* for the step-tracking task (A) and the pursuit task (B) obtained with the *searching method*. For each combination of parameters *B*_*r*_ and *K*_*r*_ (both within the physiological ranges), the correlation coefficient *R* between the muscle torque and the kinematic torque was calculated to generate a contour plot of *R* for each task. The color codes on the right side of (A) and (B) indicate the mean value of *R* for the X and Y components. The white dashed lines indicate the combination of *B*_*r*_ and *K*_*r*_ for the best *B*_*r*_ */K*_*r*_ ratios. (C) and (D) Relationship between *B*_*r*_ */K*_*r*_ ratios obtained with CCA (*absciss*a) and the *searching method* (*ordinate*) for the step-tracking task (C) and the pursuit task (D) for the controls (*green dots*) and the patients (*red dots*).

*2) Step 2*: We explored the combination of *B*_*r*_ and *K*_*r*_ that yielded the best match for both the X-axis component and the Y-axis component based on the correlation coefficient *R* for the optimization (Fig 7A and 7B).

In this study, we first tested these methods by analyzing the step-tracking task as well as the pursuit task of the wrist joint.

### Calculation of the inertia parameter *M* for the wrist joint model

We calculated an inertia parameter *M* of the hand around the wrist joint for each subject, using Eq (2), which assumes the hand to be a spherical body and the axes of rotation are on its surface. We also assumed the density of the hand to be 1.0 g/ml.

$$M=\frac{2mr^{2}}{5}+mr^{2}=\frac{7mr^{2}}{5}$$ (2)

where *m* and *r* denote the mass (kg) and the radius (m) of the hand, respectively.

### Statistical tests

We made statistical tests using two-sample t-test (*ttest2* function in the statistics toolbox of Matlab, Ver. 7.11.0.584(R2010b), Mathworks, MA) or Mann-Whitney U-test (*ranksum* function in the statistics toolbox of Matlab, Ver. 7.11.0.584(R2010b), Mathworks, MA).

## Results

### Identification of the relationship between movement kinematics and muscle activity with CCA

**Fig 8:**
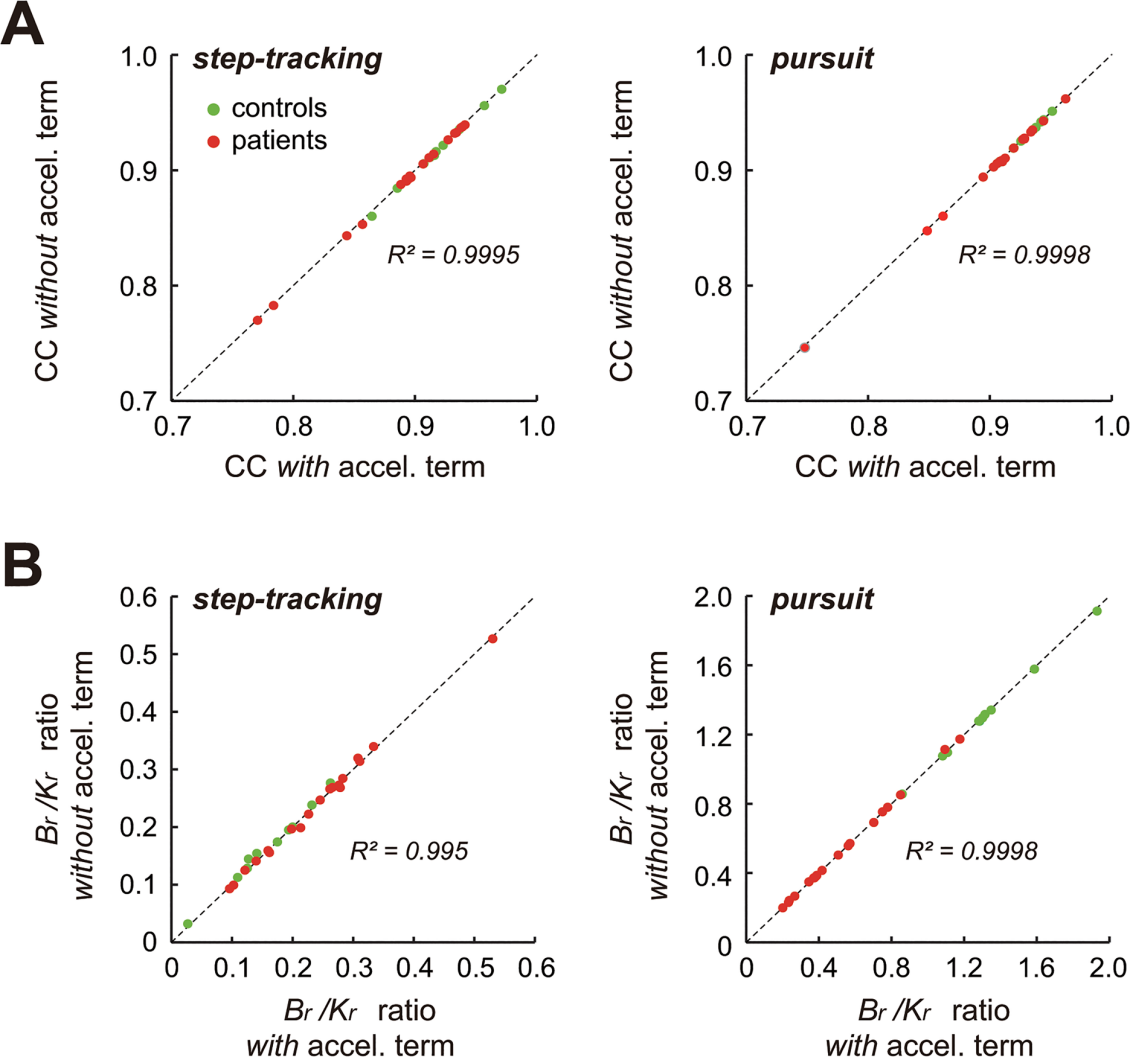
Negligible contribution of the acceleration term in the wrist joint model (1) for the two tasks. (A) *CC*’s for both the *step-tracking* task and the *pursuit* task were almost identical regardless of with or without the acceleration term in the wrist joint model (1) for both *controls* (*green dots*) and *patients* (*red dots*). Note the high values of *R* ^*2*^ between the *CC’*s with and without the acceleration term. (B) *B*_*r*_ */K*_*r*_ ratios for both the *step-tracking* task and the *pursuit* task were almost identical regardless of with or without the acceleration term in the wrist joint model (1) for both *controls* (*green dots*) and *patients* (*red dots*). Note the high values of *R* ^*2*^. Also note that the average ratio of *M*_*r*_ and *K*_*r*_ (*M*_*r*_/*K*_*r*_) obtained with *CCA* for the controls and the patients (n = 29) was 0.0048 ± 0.0036 (range: 0.0019–0.0189) for the *step-tracking* task, and 0.012 ± 0.0088 (range: 0.0032–0.0345) for the *pursuit* task, explaining why the acceleration term is negligible in the regression.

We examined the relationship between the movement kinematics of the wrist joint and the activity of the four wrist prime movers in 10 control subjects performing the two tasks of the step-tracking task (Fig 1A) and the pursuit task (Fig 1B) using the wrist joint model (1). We used CCA (see Materials and Methods) (Fig 3) to obtain the two sets of parameters *a*_*1*_, *a*_*2*_, *a*_*3*_, *a*_*4*_, (Fig 3 *left*) and *M*_*r*_, *B*_*r*_, *K*_*r*_ (Fig 3 *right*) that maximize the CC between the two canonical variates (i.e. *muscle torque* and *kinematic torque*) (Fig 3 *middle*). Fig 4A and 4B show typical examples of the relationships in the step-tracking task and the pursuit task, respectively, of a control subject. For both tasks, we obtained a precise match between *muscle torque* (i.e. (*M*_*r*_, *B*_*r*_, *K*_*r*_)($\ddot{\theta }\left(t\right),\dot{\theta }\left(t\right),\theta \left(t\right)$)^T^) (Fig 4A and 4B, red lines in the upper panel of *canonical variate*) and *kinematic torque* (i.e. (*M*_*r*_, *B*_*r*_, *K*_*r*_)($\ddot{\theta }\left(t\right),\dot{\theta }\left(t\right),\theta \left(t\right)$)^T^) (Fig 4A and 4B, blue lines in the upper panel of *canonical variate*) with high values of canonical correlation (*CC*’s) (*CC* = 0.96 for step-tracking [A]; *CC* = 0.94 for pursuit [B]). For all of the control subjects, the average *CC* was 0.92 ± 0.03 (range: 0.86–0.97, n = 10) for the step-tracking task and 0.93 ± 0.01 (range: 0.91–0.95, n = 10) for the pursuit task. Similarly, Fig 5A and 5B show corresponding examples of the relationships in a patient in the two tasks. As in the control subjects, we also found high *CC*’s for both tasks in the patient (Fig 5, *CC* = 0.94 for step-tracking [A]; *CC* = 0.95 for pursuit [B]). For all of the patients, the average *CC* was 0.89 ± 0.05 (range: 0.77–0.94, n = 19) for the step-tracking task and 0.91 ± 0.05 (range: 0.75–0.96, n = 19) for the pursuit task. In general, *M*, *B*, and *K* in Eq (1) are matrices due to the interactions between the two degrees of freedom for the wrist joint [28,29]. For simplicity, we assumed that the parameters *M*, *B*, and *K* were all scalars as Haruno and Wolpert [24] did. Indeed, our assumption was justified with the high *CC*’s (Fig 8A) between the two canonical variates (*kinematic torque* and *muscle torque*) with the two sets of scalar parameters (Figs 4 and 5). Furthermore, the two sets of parameters were sufficient to establish the high correlation for the entire duration (>13 s) of each movement task. Considering the complexity of the wrist joint biomechanics, the simple and consistent relationship between the muscle activity and the movement kinematics was unexpected.

It should be noted that we obtained three pairs of canonical variates in the present CCA, the first, the second and the third pairs of canonical variates. The average proportion of the sum of the eigenvalues for the first pair of the canonical variates was 0.98 ± 0.01 (range: 0.95–1.00, n = 29)(1.0 = 100%) for the step-tracking task and 0.95 ± 0.03 (range: 0.84–0.99, n = 29) for the pursuit task. It means that the first pair of the canonical variates explains most (~ 95%) of the data variability regardless of the task. Therefore, we consider only the first pair of canonical variates and the corresponding canonical coefficients and canonical correlation coefficients, in the following.

### Simplification of the wrist joint model

In our previous study [15], we noticed a minor contribution of the acceleration term in the identification of the pursuit task. Therefore, in this study, we evaluated the contribution of the acceleration term in both the step-tracking task and the pursuit task with CCA (Figs 4 and 5). The bottom two panels of Figs 4 (control) and 5 (patient) compare the fit results *with* (*top*) and *without* (*bottom*) the acceleration term in Eq (1). Our surprising observation was that the relationship between the movement kinematics and the muscle activities in the two tasks showed almost identical *CC’*s (Fig 8A) even when the acceleration term was omitted from the identification with CCA. In other words, the velocity term and the position term of Eq (1) dominated in the identification in both tasks. Therefore, without sacrificing accuracy, we can simplify the wrist joint model of Eq (1) to get Eq (3) by removing the acceleration term, at least for the present experimental setup.

$$\sum_{i=1}^{4}\;a_{i}\;T_{i}\left(t\right)\approx B\dot{\theta }\left(t\right)+K\theta \left(t\right)$$ (3)

### The *B*_*r*_ */K*_*r*_ ratio and its functional interpretation for motor control

In this study, it is not possible to determine the absolute values of *B*_*r*_ and *K*_*r*_, because with CCA it is only possible to determine *ratios of the fitting parameters* [25], as described before. On the other hand, it is reasonable to assume that both *B*_*r*_ and *K*_*r*_ took specific values within their physiological ranges. Therefore, we further addressed two issues: a) whether specific values of *B*_*r*_ and *K*_*r*_ provided a better correlation than other pairs of *B*_*r*_ and *K*_*r*_ with the same ratio, and b) how the correlation was altered for different combinations of *B*_*r*_ and *K*_*r*_. We explored the optimal combination of *B*_*r*_ and *K*_*r*_ that yielded the highest *R*-value for identification within the physiological ranges (Fig 6, *upper right inset*) (see also Materials and Methods) [26–28]. Fig 7 summarizes the *R*-values for different combinations of *B*_*r*_ and *K*_*r*_ for the step-tracking task (A) and the pursuit task (B) for the examples shown in Fig 4A and 4B, respectively. It was clear that the best fits for the step-tracking task (Fig 7A) and the pursuit task (Fig 7B) (represented as the highest *R*-value (i.e., the dark red region)) were obtained for the unique ratios of *B*_*r*_ and *K*_*r*_. The best fit for the step-tracking task (Fig 7A) was obtained when *B*_*r*_ was much smaller than *K*_*r*_ (dashed white lines). In contrast, for the pursuit task (Fig 7B), the best fit was obtained when *B*_*r*_ was larger than *K*_*r*_ (dashed white lines). These observations suggested that Eq (3) (or equivalently, Eq (1)) held when *B*_*r*_ was in a specific ratio to *K*_*r*_. In other words, the wrist joint torque generated by the four wrist prime movers (*middle* of Eq (3)) are de-convoluted into the two components (*right side* of Eq (3)): one is proportional to velocity and the other is proportional to the position of the wrist joint. Therefore, the ratio of *B*_*r*_ and *K*_*r*_ (we call it “*B*_*r*_ */K*_*r*_ ratio”) characterizes the muscle activities. For instance, if the muscle activities are weighted more for position control than for velocity control (i.e., *B*_*r*_ < *K*_*r*_), then the *B*_*r*_ */K*_*r*_ ratio should be less than 1.0. In contrast, if the muscle activities are weighted more for velocity control than for position control, then the *B*_*r*_ */K*_*r*_ ratio should be more than 1.0. In this way, the *B*_*r*_ */K*_*r*_ ratio provides a clue to characterize the muscle activities in terms of the movement kinematics.

### Task dependency of the *B*_*r*_ */K*_*r*_ ratio and its functional meaning for motor control in control subjects

The smaller *B*_*r*_ */K*_*r*_ ratios for the step-tracking task and the larger *B*_*r*_ */K*_*r*_ ratios for the pursuit task in the control subjects were also evident in the *B*_*r*_ */Kr* ratios obtained with CCA (Fig 8B, *step-tracking* and *pursuit*). Indeed, the ratio obtained with this method was highly correlated with the ratio obtained with CCA for both tasks (Fig 7C and 7D, *green dots*). Therefore, in the following, we refer only to *B*_*r*_ */K*_*r*_ ratios obtained with CCA. In Fig 8B, it should also be noted that the acceleration term made little, if any, contribution to the calculation of *B*_*r*_ */K*_*r*_ ratios, regardless of the task.

**Fig 9:**
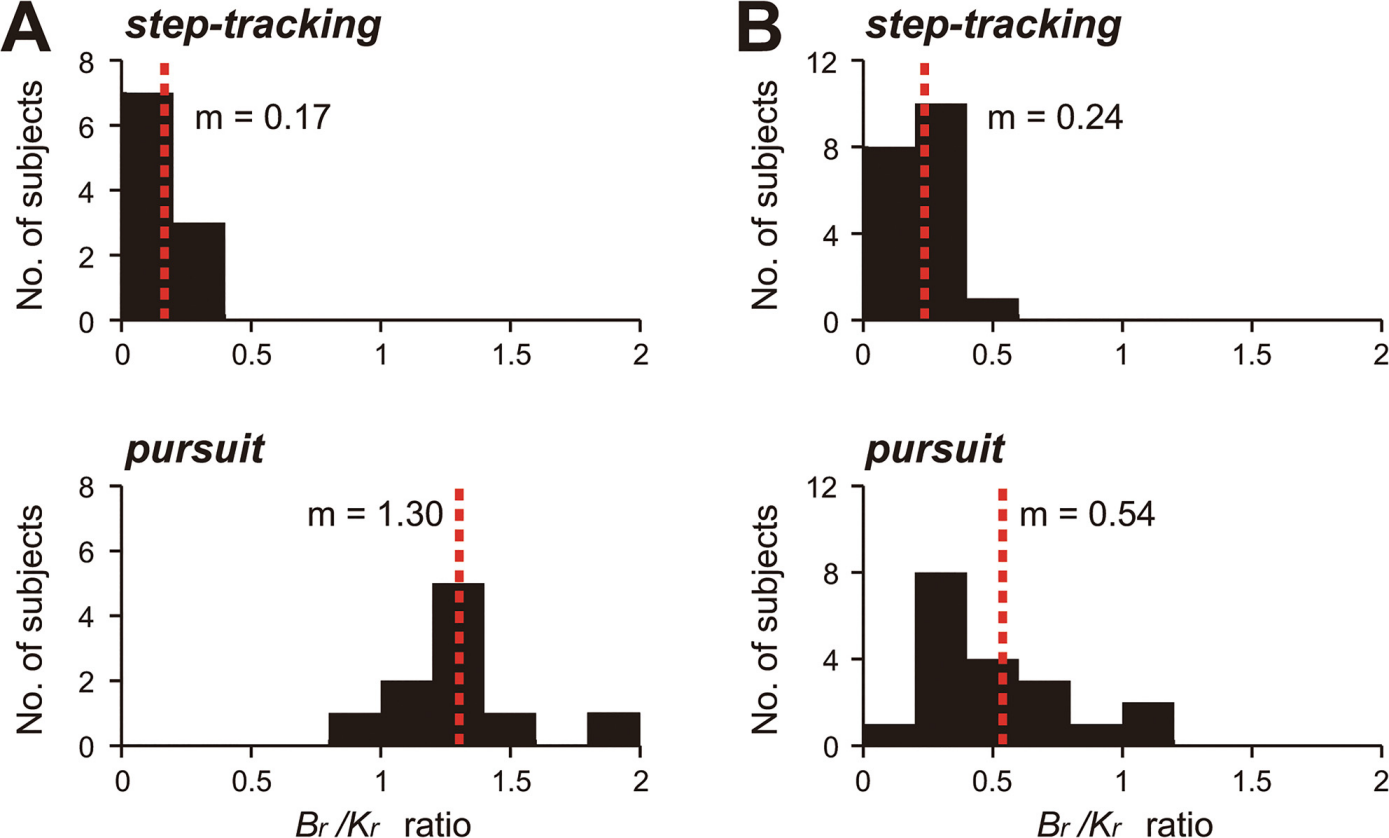
Comparison of the *B*_*r*_ */K*_*r*_ ratios for the two tasks between the controls (A) and the patients (B). (A) *B*_*r*_ */K*_*r*_ ratios of the control subjects for the step-tracking task (top) and the pursuit task (bottom) (n = 10). Note the highly significant difference in the *B*_*r*_ */K*_*r*_ ratios for the two tasks. (B) The *B*_*r*_ */K*_*r*_ ratios of the patients (Table 2, n = 19) for the two tasks (the same convention as in (A)).

For the control subjects, *B*_*r*_ */K*_*r*_ ratios for the step-tracking task ranged from 0.03 to 0.28 (mean ± standard deviation [SD] = 0.17 ± 0.06, n = 10) (Fig 9A, *step-tracking*), suggesting that the muscle activities for the step-tracking task were weighted more for position control than for velocity control of the wrist joint. In contrast, the *B*_*r*_ */K*_*r*_ ratios calculated with CCA for the pursuit task were much higher, ranging from 0.86 to 1.91 (mean ± SD = 1.30 ± 0.27, n = 10) (Fig 9A, *pursuit*). Thus, the muscle activities for the pursuit task correlated comparably with both, the velocity and position of the wrist joint.

**Fig 10:**
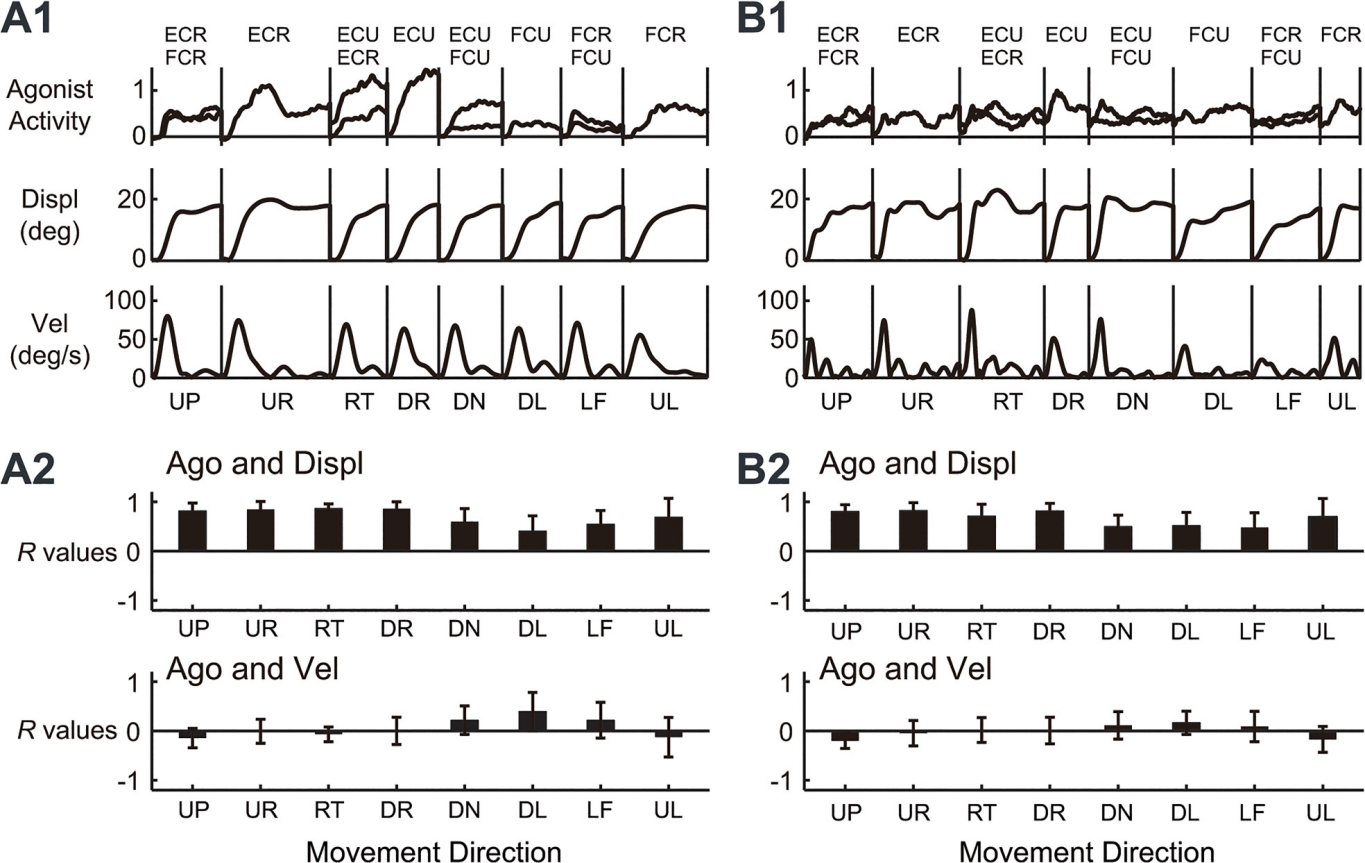
Similarities between the agonist muscle activity and wrist position in the step-tracking task. (A1) Comparison of agonist muscle activity (*Agonist activity*), displacement (*Displ*), and tangential velocity (*Vel*) of the wrist joint for the eight directions for the control subject shown in Fig 4A. Note the similarity of *Agonist* to *Displ*, regardless of the movement direction. (A2) Correlation coefficient (*R* values) for *Agonist* (*Ago*) and *Displ* or *Ago* and *Vel* for all of the control subjects. For the directions with two agonists, we calculated the mean *R* for the two muscles. The data from 10 control subjects were averaged. For the abbreviations of direction and muscle name, see Figs 1 and 2, respectively. (B1) The same convention as in (A1) for the data from the patient shown in Fig 5A. (B2) The same convention as in (A2) for the data from the 19 patients with cerebellar ataxia.

One interpretation of these observations was that, when generating commands for a step-tracking task to a fixed target, the central nervous system (CNS) primarily considers the target position. In fact, we directly compared the activity pattern of the agonist muscle(s) with the position (displacement) and velocity (tangential velocity) of the wrist joint for the step-tracking task of the normal control shown in Fig 4A (Fig 10 A1). Apparently, the temporal patterns of the activities of the agonist muscles were far more similar to displacement than to velocity for almost all of the directions. Indeed, for all control subjects, the activities of the agonist muscles showed higher correlations with position (displacement) than with velocity (Fig 10 A2, n = 10, *p* < 0.05 for all directions except for the DL direction, Student’s *t*-test). In other words, the muscle activities in the step-tracking task contained much more of a component for position control than for velocity control. In contrast, for the pursuit task, the target moved with predetermined velocity and position. Thus, the CNS must control both velocity and position to optimally pursue the moving target [30–32]. In fact, in Fig 4B, the temporal patterns of the estimated canonical variates (i.e. wrist joint torque) (*bottom*) were intermediate between the position (*X*) and the velocity (*Vx*). Overall, the optimal *B*_*r*_ */K*_*r*_ ratio was task-dependent and appeared to reflect a control strategy of the CNS for the requirement of each task.

### Functional differences in the muscle activities for two tasks and its application for a quantitative evaluation of cerebellar ataxia

Next, with the same CCA, we determined the *B*_*r*_ */K*_*r*_ ratios of the patients with cerebellar ataxia for the two tasks and compared them with those of the control subjects. Fig 9B summarizes the distributions of the *B*_*r*_ */K*_*r*_ ratios of the patients for the two tasks. For the step-tracking task (Fig 9B, *step-tracking*), the *B*_*r*_ */K*_*r*_ ratios of the cerebellar patients (range: 0.09–0.53, mean ± SD = 0.24 ± 0.10, n = 19, p > 0.08, Mann-Whitney *U* test) were not different from those of control subjects. In accordance with the *B*_*r*_ */K*_*r*_ ratios, the temporal patterns of activities of the agonist muscles for the patient shown in Fig 5A were far more similar to displacement than to velocity for all directions, as in the control subjects (Fig 10 B1). Indeed, in all patients with cerebellar ataxia, the activities of the agonist muscles showed higher correlations with position (displacement) than with velocity (Fig 10 B2, n = 19, *p* < 0.001 for all directions, Student’s *t*-test). In summary, the muscle activities in the step-tracking task contained a much greater component for position control than for velocity control in both, the patients and control subjects. However, for the pursuit task, the *B*_*r*_ */K*_*r*_ ratios of patients (Fig 9B, *pursuit*, range: 0.20–1.17, 0.54 ± 0.28, n = 19) were much smaller than those of the control subjects (Fig 9A, *pursuit*, range: 0.86–1.91, 1.30 ± 0.27, n = 10), and this difference was highly significant (*p* < 4.9 × 10^−5^, Mann-Whitney *U* test).

In summary, separation of the *B*_*r*_ */K*_*r*_ ratios for the two tasks was altered systematically depending on the existence of cerebellar ataxia (Fig 9A and 9B). For the control subjects, the distributions of the *B*_*r*_ */K*_*r*_ ratios for the two tasks were clearly separated without overlap (Fig 9A). In contrast, for the cerebellar patients, the distributions of the *B*_*r*_ */K*_*r*_ ratios largely overlapped (Fig 9B).

It should be noted that the task-dependent changes in the *B*_*r*_ */K*_*r*_ ratios in the control subjects were compatible with the requirements of the individual tasks, as mentioned previously. In other words, the control subjects could switch the composition of the motor commands from one mode to another adaptively. However, for cerebellar patients, only the position-dominant mode, which could be a default mode, was available. Overall, the difference in the *B*_*r*_ */K*_*r*_ ratios for the two tasks provides a useful quantitative parameter to characterize motor control function in patients in terms of the composition of the motor commands (Fig 9).

## Discussion

In this study, we compared the functional characteristics of muscle activities for two movement tasks, a step-tracking task and a smooth pursuit task. We proposed a simple linear model for the wrist joint that characterized the causal relationship between muscle activities and movement kinematics (specifically, position and velocity) in human subjects. We found that, in control subjects, the CNS adjusted the composition of the motor commands (i.e., the muscle activities) to meet the task requirement. For instance, for the step-tracking task toward stationary targets, the muscle activities were correlated primarily with the wrist position, with little consideration of velocity (i.e., low *B*_*r*_ */K*_*r*_ ratio; Fig 9A, *step-tracking*). However, for the smooth pursuit task, in which the target moves with known velocity and position, the muscle activities were correlated both with the velocity and position of the target (i.e., higher *B*_*r*_ */K*_*r*_ ratio; Fig 9A, *pursuit*). In contrast, the ability of patients to adjust the motor commands (i.e., select a proper *B*_*r*_ */K*_*r*_ ratio) deteriorated with cerebellar ataxia (Fig 9B). Therefore, the ability to switch between different control strategies provides a novel quantitative parameter to characterize the motor function of patients with cerebellar ataxia.

### Functional interpretation of the *B*_*r*_ */K*_*r*_ ratio

In order to determine *B*_*r*_ and *K*_*r*_, it is necessary to measure the wrist joint torque τ(t). However, τ(t) was not measurable in the present study, making it impossible to determine the absolute values of *B*_*r*_ and *K*_*r*_. Instead, we used CCA to find the optimal ratio of *B*_*r*_ and *K*_*r*_ that yielded the best match between the two canonical variates, i.e. the muscle torque (*middle*) and the kinematic torque (*right side*). We found that the best match was obtained when *B*_*r*_ was in a specific ratio to *K*_*r*_, and the ratio varied systematically for each task (Fig 7). It should be emphasized that the inertia term (i.e. $M_{r}\ddot{\theta }$) had little impact on the regression for both tasks (Figs 4, 5, 6 and 8A), regardless of the method employed, thereby suggesting its negligible contribution in the present experimental setup.

In other words, the wrist joint torque that was generated by the four wrist prime movers was mostly explained as sum of the two components: the velocity term (i.e. $B_{r}\dot{\theta }$) and the position term (i.e. *K*_*r*_ *θ*). With this view, the parameters *B*_*r*_ and *K*_*r*_ can be interpreted as the weights for the two components, and these determined the temporal profile of the wrist joint torque. For instance, if the controller set the weight *B*_*r*_ much higher than the weight *K*_*r*_, the temporal pattern of the wrist joint torque should be more similar to the velocity rather than to the position of the wrist joint. In contrast, if the controller set the weight *K*_*r*_ much higher than the weight *B*_*r*_, the temporal pattern of the wrist joint torque should be more similar to the position of the wrist joint. In other words, the ratio of *B*_*r*_ and *K*_*r*_ indicates the composition of the motor command generated by the controller. Indeed, for the step-tracking task, in which the target was stationary without explicit reference velocity (Fig 1A), the *B*_*r*_ */K*_*r*_ ratio in control subjects was low, ranging from 0.03 to 0.28 (mean ± SD = 0.17 ± 0.06, n = 10; Fig 9A, *step-tracking*). This suggested that the muscle activities for the step-tracking task were weighted more for position control than for velocity control. Here, we called this type of control *position-dominant* control. We confirmed that the activities of agonist muscles during the step-tracking task showed higher correlations with position (displacement) than with velocity for all control subjects (Fig 10A). However, for the pursuit task in which the target moves with known velocity and position, both position and velocity were key control parameters to keep the cursor within the moving target (Fig 1B). The *B*_*r*_ */K*_*r*_ ratio for the pursuit task was much higher, ranging from 0.86 to 1.91 (mean ± SD = 1.30 ± 0.27, n = 10; Fig 9A, *pursuit*). This indicated that the muscle activities for the pursuit task were weighted comparably with both the velocity and position of the wrist joint. We called this type of control *velocity/position* control. Indeed, the temporal patterns of muscle torque appeared intermediate between the position and the velocity (see Fig 4B).

In general, *B* and *K* are dependent on the level of muscle activity [33,34]. Thus, we expected dynamic changes in the optimal combination of *B*_*r*_ and *K*_*r*_ during the trial. On the contrary, we observed that the *B*_*r*_ */K*_*r*_ ratio was stable for the entire duration of the tasks (Fig 4). In other words, the controller in the CNS somehow set *B*_*r*_ and *K*_*r*_ in a specific ratio (*B*_*r*_ */K*_*r*_ ratio) for each task. Overall, the *B*_*r*_ */K*_*r*_ ratio appeared to be a useful parameter to represent a feature of the motor command.

### *B/K* ratios in previous studies

Some researchers have performed complicated experiments to estimate the real values of *B* and *K* for the wrist joint during visually guided step-tracking tasks with parametric system identification and a second-order linear equation of motion [35,36]. For instance, Grey [36] estimated *B* and *K* to be 0.03 Nms/rad and 6.3 Nm/rad, respectively, and, therefore, the *B/K*ratio (= 0.03/6.3) was 0.005. Similarly, Milner and Cloutier [35] estimated *B* and *K* for flexion to be 0.123 Nms/rad and 9.22 Nm/rad, respectively, and *B* and *K* for extension to be 0.094 Nms/rad and 4.08 Nm/rad, respectively. Specifically, the *B/K* ratios were 0.013 for flexion and 0.023 for extension. Overall, the *B/K* ratios for the step-tracking tasks of the wrist joint in previous studies are comparable to or even lower than those obtained in the present study (range: 0.03–0.28, mean ± SD = 0.17 ± 0.06, n = 10; Fig 8C). The low *B/K* ratios of the previous studies are expected because they required subjects to control only position. However, other studies have estimated *B* and *K* for the relaxed wrist joint [28,37–39]. The latter studies estimated *B* to range from 0.02 to 1.05 Nms/rad, while estimated *K* ranged from 0.32 to 36.7 Nm/rad. Consequently, the *B/K* ratios for the relaxed wrist joint ranged from 0.005 to 0.09 and were comparable to or even lower than the *B*_*r*_ */K*_*r*_ ratios for the step-tracking task. However, it should be noted that their results were obtained in a passive condition without active muscle activity. Therefore, functional interpretations of the low *B*_*r*_ */K*_*r*_ ratios in these experiments need to be addressed in a future study.

### Comparison of the *B/K* ratio with the damping ratio

In the field of biomechanics, the damping ratio ζ (4) provides a convenient measure to quantify the level of damping (i.e., viscosity *B*) relative to the critical damping ($2\sqrt{KM}$) [27,40–42]. By modifying Eq (4) into Eq (5), it is clear that the damping ratio ζ has a direct relationship to the *B*_*r*_ */K*_*r*_ ratio introduced in this study.

$$\zeta =\;B/2\sqrt{KM}$$ (4)

$$\zeta =\;\alpha \cdot B/\sqrt{K}\;(\alpha =1/2\sqrt{M}\;:\;\text{constant})$$ (5)

According to Eq (5), the damping ratio and the *B*_*r*_ */K*_*r*_ ratio correlate with each other. In general, when ζ is less than 1 (ζ < 1), the system is considered *underdamped*. In contrast, when ζ is larger than 1 (ζ > 1), the system is considered *overdamped* [40]. For the identification shown in Fig 4A, ζ for the step-tracking task in the control subject was 0.55, suggesting that the wrist joint was underdamped. In previous studies, some researchers have estimated ζ for the voluntary movements of various joints. For instance, Milner and Cloutier [27] reported that ζ ranged from 0.3 to 0.4 during flexion movements of the wrist joint. For the elbow joint, Bennett et al. [41] reported that ζ ranged from 0.2 to 0.6 during voluntary sinusoidal motion. Comparable ζ’s (0.2–0.4) were reported for abduction/adduction of a finger joint [42]. The underdamped state (ζ < 1) in these previous studies was expected because their tasks required only position control (i.e., low *B*_*r*_ */K*_*r*_ ratio), as was the case for the step-tracking task used in the present study. In contrast, ζ for the pursuit task of the same control subject (Fig 4B) was 4.54, suggesting that the wrist joint was highly overdamped. Overall, in the control subjects, the wrist joint was underdamped (ζ < 1) for the position-dominant control of the step-tracking task (i.e., lower *B*_*r*_ */K*_*r*_ ratio), while it was overdamped (ζ > 1) for the velocity-dominant control of the pursuit task (i.e., higher *B*_*r*_ */K*_*r*_ ratio).

### The *B*_*r*_ */K*_*r*_ ratios for two movement tasks in patients with cerebellar ataxia and the role of the cerebellum in velocity and position contro*l*

The *B*_*r*_ */K*_*r*_ ratios in the control subjects were task-dependent (Fig 9A) and appeared to reflect a change in the control strategy of the CNS for different tasks. In contrast, in the patients, the task-dependent changes in the *B*_*r*_ */K*_*r*_ ratios were much smaller (Fig 9B). Indeed, patients used position-dominant control even for the pursuit task (Fig 9B, *pursuit*). In other words, they were not able to switch to the velocity-dominant control. Similarly, the damping ratios ζ’s for the two tasks failed to show task-dependent changes. The damping ratio ζ for the step-tracking task of the patient with cerebellar ataxia (Fig 5A) was 1.05, while the ζ for the pursuit task of the same patient (Fig 5B) was 1.20. It should be noted that the slightly overdamped conditions (i.e., ζ > 1) for both tasks reflected increased levels of co-contraction in the patients (unpublished observation) to reduce instability. Overall, these findings suggested that the cerebellum makes an important contribution to generating muscle activities for velocity control. These observations also explain why cerebellar ataxia is characterized by a lack of smoothness. Patients have to substitute a smooth movement, which requires finely graded velocity control, with *intermittent* step-wise movements, which are manageable with simple position control. The step-wise position-control movement appears to be a default and primitive mode of motor control that is available for patients with cerebellar ataxia. Continuous velocity control is predictive in nature. Therefore, the impaired velocity control in these patients may suggest a problem of predictive control in cerebellar deficits. The prediction may consist of two components: 1) prediction of target motion; 2) prediction of self-movement. The former is more cognitive than the latter. Unfortunately, the pursuit task used in the present study is not designed to dissociate the two components. We are going to address this issue in our future study.

It should be noted that our results do not necessarily mean that the cerebellum is *not* important in position control. As shown in Fig 5A, the inaccuracy of the step-tracking task strongly suggested that position control was also impaired in patients with cerebellar ataxia. Therefore, the cerebellum is also deeply involved in position control, probably through agonist selection and/or determining the proper timing and ratio of the activities of agonists and antagonists [1,8–11]. Overall, it is likely that the cerebellum plays an essential role in generating precise temporal patterns of muscle activities for both velocity and position controls. Our results might also suggest that the cerebellum is important for changing the mode of muscle activity depending on the task requirements. Our results were consistent with those of neurophysiological studies in which the activities of Purkinje cells correlate with the position and velocity of arm movements in monkeys [43–45].

### Quantitative evaluations of the motor functions of patients with cerebellar ataxia based on the *B*_*r*_ */K*_*r*_ ratio

Quantitative evaluations of motor functions of patients are essential for monitoring the progress of disease and evaluating treatment effects. To date, some researchers have tried to perform quantitative evaluations of cerebellar ataxia with arm movements [6,7,46]. However, their evaluations were mostly limited to movement kinematics. They captured some features of movement disorders, such as more curved and less smooth hand paths, with a more asymmetric speed profile, in patients with cerebellar disease. However, movement kinematics cannot determine causal muscle activities or motor commands due to the redundancy of the musculoskeletal system. In order to precisely evaluate the condition of the patients, it is desirable to capture anomalies of the motor commands directly [10,11] rather than the resultant movement anomalies. In this study, we evaluated the motor functions of patients with cerebellar ataxia based on the motor command levels with EMG signals. In particular, the decreased *B*_*r*_ */K*_*r*_ ratio for pursuit task appeared to reflect the pathophysiological changes in these patients (Fig 9B). We are currently testing this hypothesis by repeatedly evaluating the *B*_*r*_ */K*_*r*_ ratios for pursuit task in patients with cerebellar ataxia for a long period.
